# Ensuring Reproduction at High Temperatures: The Heat Stress Response during Anther and Pollen Development

**DOI:** 10.3390/plants2030489

**Published:** 2013-07-11

**Authors:** Filomena Giorno, Mieke Wolters-Arts, Celestina Mariani, Ivo Rieu

**Affiliations:** Department of Molecular Plant Physiology, Radboud University Nijmegen, Heyendaalseweg 135, 6525 AJ Nijmegen, The Netherlands; E-Mails: mwolters@science.ru.nl (M.W.-A); c.mariani@science.ru.nl (C.M.); i.rieu@science.ru.nl (I.R.)

**Keywords:** heat stress, pollen development, male sterility, fruit set, tomato

## Abstract

Sexual reproduction in flowering plants is very sensitive to environmental stresses, particularly to thermal insults which frequently occur when plants grow in field conditions in the warm season. Although abnormalities in both male and female reproductive organs due to high temperatures have been described in several crops, the failure to set fruits has mainly been attributed to the high sensitivity of developing anthers and pollen grains, particularly at certain developmental stages. A global view of the molecular mechanisms involved in the response to high temperatures in the male reproductive organs will be presented in this review. In addition, transcriptome and proteomic data, currently available, will be discussed in the light of physiological and metabolic changes occurring during anther and pollen development. A deep understanding of the molecular mechanisms involved in the stress response to high temperatures in flowers and, particularly, in the male reproductive organs will be a major step towards development of effective breeding strategies for high and stable production in crop plants.

## 1. Introduction

Higher plants have a complex life cycle in which growth of the diploid sporophytic organism alternates with a gametophytic haploid form. As sessile organisms, plants are often at the mercy of environmental factors such as drought, cold, salinity and high temperatures, which can markedly affect the success of reproduction and fertilization processes. Although the cultivated crops are widely adapted to grow in many areas of the world, some developmental stages are very sensitive to environmental stresses, particularly temperature fluctuations occurring during anther and pollen development [1,2,3,4]. Such vulnerability is considered an important factor for the failure to set fruits under high temperatures [5,6]. Taking into account the recent projection of global warming, significant efforts are currently devoted to develop crops with improved thermotolerance.

In response to high temperatures, plants manifest different physiological adaptations, including short-term stress avoidance or acclimatization mechanisms involving leaf re-orientation, cooling by transpiration, or alteration of membrane lipid compositions [7]. A common feature of the plant response is that an initial exposure to moderately elevated temperature provides resistance against a subsequent usual lethal dose of heat stress (hs). This phenomenon is referred to as acquired thermotolerance [7,8]. Even when plants grow in their natural distribution range, they may experience high temperatures or diurnal fluctuations that would be lethal in absence of this rapid acclimatization. Therefore, the acquisition of the thermotolerance may reflect a general mechanism that contributes to the homeostasis of the metabolism on a daily basis [7]. At the cellular level, plants manifest different biochemical and physiological adjustments and a cascade of events, which start with stress perception and end with the expression of a specific set of responsive genes [9,10]. Due to the fact that not all processes taking place in a plant are equally sensitive to high temperatures [7], thermotolerance can be developmentally and tissue-specifically regulated. The elucidation of this developmentally regulated thermotolerance will facilitate the production of crop cultivars able to cope with hs at crucial times during their growth. 

Here, we will present an overview of the impact of hs on male reproductive development in tomato, an important crop species, often exposed to temperature fluctuations during flowering.

## 2. Heat Stress Effects on Developing Pollen Grains and Anther Tissues

**Figure 1:**
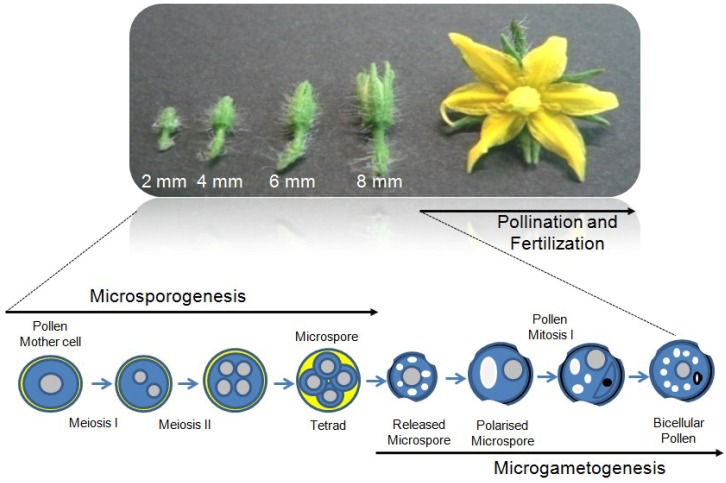
Tomato flower buds at different developmental stages from cv Saladette. Young buds ranging from 2 mm to 8 mm in length correspond to the phases in which microsporogenesis and microgametogenesis processes occur in anthers, while pollination and fertilization take place at anthesis stage.

Male gametophyte development takes place in specialized organs of the flowers, the anthers, consisting of epidermis, endothecium, tapetum and connective tissues surrounding the sporogenous cells [11,12]. Single determined sporophytic cells, the archespores corresponding to the male germline, are committed to produce gametes by two sequential distinct phases, namely microsporogenesis and microgametogenesis (Figure 1). Microsporogenesis is characterized by the meiotic division of diploid pollen mother cells to produce tetrads of haploid microspores, which are then released as uni-cellular microspores after activity of a mixture of enzymes secreted by the tapetum layer [13]. During microgametogenesis, the polarized released microspores enlarge and a single large vacuole is produced [14,15,16]. This is accompanied by migration of the microspore nucleus to a peripheral position against the microspore wall. The microspores then undergo an asymmetric cell division known as Pollen Mitosis I (PMI). The small germ cell, called the generative cell, is subsequently engulfed within the cytoplasm of the larger vegetative cell to create a novel cell-within-a-cell structure. Vegetative and germ cells have distinct fates. 

The vegetative cell nurtures the germ cell and gives rise to the pollen tube following successful pollination. In tomato, during pollen tube growth in the stylar tissue, the germ cell goes through a further round of mitosis, called Pollen Mitosis II (PMII), to produce twin sperm cells. This process already occurs in the pollen grains in species like Arabidopsis, which shed tri-cellular pollen. Several types of metabolites accumulate in the vegetative cell during pollen maturation including carbohydrates and/or lipids, along with transcripts and proteins, which are then required for rapid pollen tube growth [17]. Osmoprotectants, including disaccharides, proline and glycine-betaine, which are thought to protect vital membranes and proteins from damage during dehydration, also accumulate [18].

**Figure 2:**
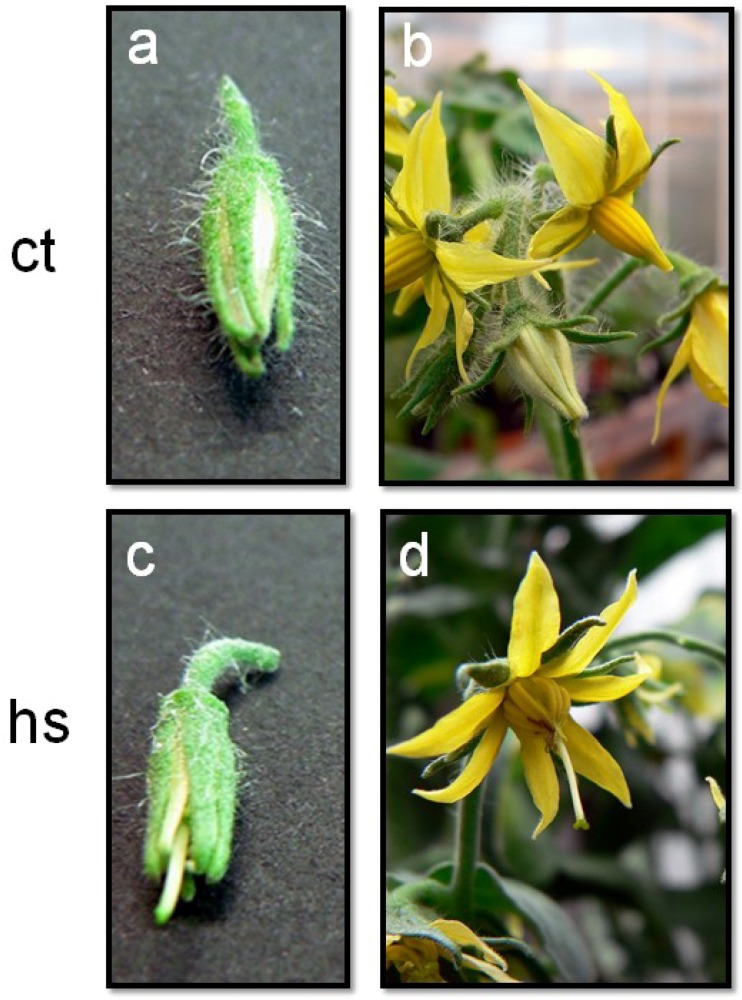
Phenotypic alterations in tomato flowers exposed to prolonged heat stress (hs) conditions. (**A**,**B**) Young flower buds of 8 mm in length and flowers at anthesis stage harvested from tomato plants (cv Saladette) grown under normal temperature (ct: 26 °C/19 °C; day/night). (**C**,**D**) Flower buds and opened flowers from plants exposed to prolonged high temperature conditions (hs: 36 °C/26 °C; day/night). Abnormal anthers and style elongation are observed in flowers treated with hs.

Temperature fluctuations occurring during the flowering period can induce morphological changes in flower organs. These changes are dependent on the intensity (temperature degrees), duration and rate of the temperature increase. Figure 2 shows some phenotypic alterations induced in tomato flowers exposed to prolonged high temperature of 36 °C. Heat stress alterations were observed in young buds and flowers at anthesis stage and were characterized by abnormal anthers and style elongation.

During reproductive development in various species, processes reported to be very sensitive to hs include meiosis in both male and female organs, pollen germination, pollen tube growth, pollen/pistil interactions, fertilization and post-fertilization processes, formation of the endosperm and embryo development [1,2,3,4]. However, within these processes, some stages are more sensitive than others. In Arabidopsis short periods of hs specifically affect pollen development during meiosis [19]. Similarly, in barley, chronic mild hs at 30 °C/25 °C for 5 days results in the failure of tapetum differentiation and injuries to the microsporogenesis process [20]. In tomato, a dramatic decrease in fruit set in response to hs has mainly been attributed to the impairment of pollen development occurring in anthers 8–13 days before anthesis, corresponding to the shift from pollen mother cells to uni-cellular microspores [3,21]. This hs sensitivity of the male reproductive organs has been observed after short episodes of high temperatures at 40 °C and after chronic exposure to 32 °C/28 °C (day/night), which is just few degrees above the optimal physiological growth range of 26 °C/22 °C (day/night) [21].

**Figure 3:**
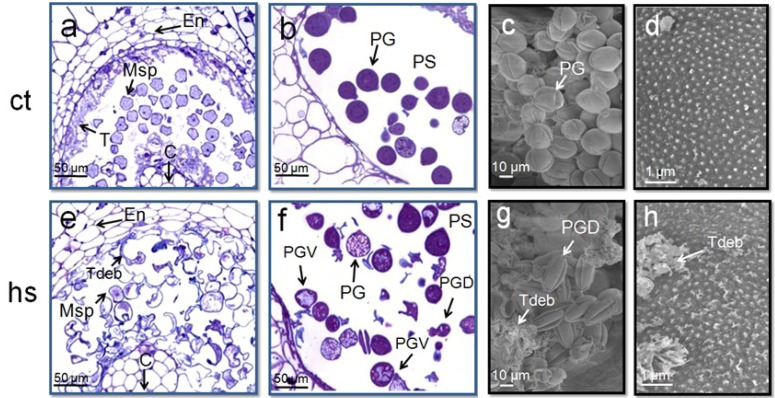
Cytological alterations in tomato anthers and pollen grains after 3 days of mild hs of 36 °C. Panels (**A**,**B**,**E**,**F**) show sections of anthers from young flower buds (**A**,**E**) and pollen grains from flowers at anthesis stage (**B**,**F**) harvested from tomato plants (cv Saladette). (**A**) Anthers from plants grown under control conditions (ct: 26 °C/19 °C; day/night) with normally developed endothecium, tapetum and microspores. (**E**) Similar anthers from plants grown at high temperature (hs: 36°C/26°C; day/night) in which tapetum and most of microspores are degenerated. (**B**) Normally developed pollen grains with densely stained cytoplasm in pollen sac. (**F**) Pollen sac of anthers treated with hs in which pollen grains are degenerated or show abnormal vacuolization. (C,D,G,H) Scanning electron micrographs of mature pollen grains. (**C**,**D**) Well hydrated pollen grains and the exine with tiny spinules, respectively, from plants grown at normal temperatures. (**G**,**H**) Not hydrated pollen grains and tapetum debris (**G**) and an irregular exine surface with tapetum debris (H) from pollen grains in anthers treated with hs.

Male sterility induced by high temperature appears to be associated with several morphological alterations of the sporophytic anther tissues such as tapetum, epidermis, endothecium and stomium [3,22,23]. We confirm the presence of striking alterations in sporophytic and gametophytic tissues of tomato after exposure to 3 days at 36 °C/26 °C (day/night, Figure 3). Most of the significant changes typically occur in the tapetum layer, but also in mature microspores that show alterations in the vacuolization (Figure 3). Similar alterations have been reported in other crop species [20,24]. Because the tapetum normally secretes callase to release the microspores from the tetrad wall and serves as a nutritive source during microspore development, the hs-induced tapetal defects may strongly affect the progression of male gametogenesis and, therefore, the correct formation of microspore cells as reported in male sterile mutants [12,13,20,25].

En: endothecium; T: tapetum; C: connective tissue; Msp: microspores; PG: mature pollen grain normally developed, PS: pollen sac; Tdeb : Tapetum debris; PGD: degenerated pollen grain; PGV: abnormal pollen grains.

## 3. Transcriptional Adjustments in Male Reproductive Organs in Response to High Temperatures

A large number of transcriptome analyses have been undertaken to shed light on the genes involved in the essential steps of pollen formation and the cooperative interactions between gametophytic and sporophytic tissues within the anthers [11,13,16]. Progress in this area was especially rapid due to the development of various techniques, such as microarray platforms and, more recently, high throughput next generation sequencing [26,27]. Improvement of suitable techniques for targeted isolation of male gamethophytic cells, based on micromanipulation, fluorescence-activated cell sorting and laser-assisted microdissection, have considerably facilitated the application of high throughput technologies on developing pollen grains and anther tissues [16,28,29,30,31]. The majority of these studies were conducted in model species such as Arabidopsis and rice, for which web-accessible resources for browsing and retrieving knowledge on genes related to plant male reproduction are currently available [32]. For tomato, high quality transcriptome analyses of developing anthers and/or pollen grains are still lacking. Recent application of high-throughput RNA-seq identified 22,000 annotated loci expressed in Arabidopsis at the stage of the meiosis [33]. However, the first studies on pollen transcriptome in Arabidopsis led to the identification of 14,000 genes associated with the development from the uni-cellular microspores to mature pollen grains [28,34,35,36], with major changes in transcript profiles and diversity observed from uni-cellular to bi-cellular microspores [28,35,36]. This shift in transcript abundance and diversity is likely associated to the fact that bi-cellular pollen has almost completed the differentiation, and mature pollen grains mostly store pre-synthesized mRNAs and proteins [16,37]. Comparisons between different databases have also provided important indications about overlap in gene expression activities between gametophytic and sporophytic transcriptomes, and male gametophyte specific genes were identified. In Arabidopsis these genes are mostly involved in pollen germination and tube growth [16,28,29,35]. Similarly to Arabidopsis, a decreased diversity of transcripts in developing rice pollen was observed from uni-cellular microspores to mature pollen grains [29]. Interestingly, developing rice pollen grains at bi-cellular and mature stages appeared to express more stage-enriched transcripts associated with defense/stress, signaling, transcription and RNA processes than those of Arabidopsis, suggesting possibly important differences between the eudicot and monocot plant lineages [29]. 

Few investigations have examined the gene expression profiles occurring in the male reproductive organs upon hs, mainly by applying microarray techniques (Table 1). Results retrieved from such analyses indicate that a large number of genes is differentially expressed in response to hs in the male reproductive organs (Table 1). Even though the transcriptome from different crops cannot be directly compared due to fact that different experimental setups and microarray platforms were used, remarkably fewer transcriptome modulations after hs were observed in tomato as compared to other crops [38,39,40]. Male reproductive cells undergo both general and specific transcriptional alterations in response to temperature changes [24,39,40,41]. As observed in vegetative tissues, the general transcriptional changes include a rapid reprogramming of cellular activities by the activation of genes related to stress perception, downstream signaling events and transcriptional controls, which in turn activate stress responsive mechanisms to re-establish homeostasis and protect and repair proteins and membranes [24,42]. 

Specific transcriptional changes involved silencing of cell proliferation and DNA replication genes as well as of genes predominantly expressed in tapetum cells, particularly those encoding for enzymes with hydrolytic functions [24,41,42,43]. In hs barley anthers, a premature activation of the meiosis specific gene *Asy1* and genes coding for anther lipid transfer proteins (LTP) was reported [24]. The latter genes are normally highly expressed in mature tapetum cells just prior to degenerations [13]. It was thus hypothesized that high temperature causes an acceleration of early anther developmental programs, such as a progression of pollen mother cells into meiosis, and cell proliferation arrest and cell degradation in the anther wall. Altogether, these events might then be responsible for the hs-induced pollen abortion and male sterility.

**Table 1:** Survey of gene expression experiments performed with male reproductive organs under hs.

Tissues	Species	Transcriptome profiling methods	Number of genes differentially expressed	References
Anther at the stage of pollen mother cells in meiotic division	*Barley*	22K Barley1 GeneChip	3353	[24]
Anthers during microsporogenesis	*Rice*	Agilent 22K rice oligo Microarray	1185	[41]
Anthers at the stage of meiosis	*Rice*	4×44K rice oligo	2449	[42]
Panicle at the 5-leaf stage corresponding to pollen mother cells undergoing meiotic division	*Barley*	SAGE libraries	31	[43]
Developing microspores at post-meiotic stages, vacuolated and early binucleate microspores	*Tomato*	cDNA-AFLP Affymetrix GeneChip	104	[38]
Developing meiotic anthers	*Tomato*	cDNA-AFLP 90K Custom Tomato Array 1.0chip	181	[39]

Phytohormones such as gibberellins, auxin and ethylene play an important role during pollen development [44,45,46,47,48] and recent data point to their involvement in the protection of male reproductive cells exposed to hs. Auxin is produced in both early and late pollen developmental stages and regulates the progression of the microspores into the mitotic divisions, filament elongation, pollen maturation and anther dehiscence [44,45,49]. In barley anthers exposed to chronic hs, a decrease in auxin concentration appears to be a major factor responsible for pollen abortion and sterility [50]. Similarly, reduction of ethylene levels increases the sensitivity of tomato microspores to moderately high temperatures [51]. 

## 4. Hsf and Hsp: Key Components in the hs Response in Anthers and Developing Pollen Grains

There is a considerable amount of evidence indicating that heat stress transcription factors (Hsfs) and heat shock proteins (Hsps) are key components in the molecular machinery activated in response to hs [9,10,52,53,54,55]. Therefore, particular attention was given to the Hsf and Hsp transcriptional modulations in pollen and anther development upon hs. High temperature sensitivity of male reproductive cells during development has been often attributed to a weak capability of pollen grains to mount an hs response and, consequently, to produce large quantities of Hsps, as compared to vegetative tissues [56]. This inability was hypothesized to be due to low or missing expression of Hsfs. Hsf proteins recognize and bind specific conserved palindromic motifs, the heat shock elements (HSE: 5'-nGAAnnTTCnnGAAn-3'), present in the promoters of the target genes, including those encoding for the Hsps [52,57]. However, this hypothesis may only apply to mature pollen, because different investigations have revealed high expression of Hsps in developing anthers and pollen grains upon hs [24,38,39,42,58,59]. These Hsps belong to different classes, including Hsp100, Hsp90, Hsp70, Hsp60 as well as small Hsps in the range of 17–30 kDa [9,53]. Hsps, as molecular chaperones, typically bind denatured proteins formed after hs, and protect them against misfolding, aggregation or premature clearance, and enable the refolding and the restoration of the native conformations [60,61,62,63,64]. Therefore, elevated levels of Hsps in male reproductive cells may protect them from cytotoxic conditions and participate in the establishment of protection and thermotolerance mechanisms, as they do in vegetative tissues [58,59,60,61,62,63,64]. Concomitantly with the presence of Hsps, transcripts encoding Hsf proteins were also found in male reproductive organs under hs. In rice panicles, eight Hsfs, of which five belonging to the class A (HsfA2a, HsfA2d, HsfA2f, HsfA9, HsfA3) and three belonging to class B (HsfB2a, HsfB2b, HsfB2c), exhibited early-up or constitutive-up-regulations during hs treatment [42]. Elevated levels of HsfA2 and HsfA3 were also detected in pollen grains of tomato from the stage of pollen mother cell to mature microspores [38,59]. Recently, a genome-wide expression analysis of all Hsfs in tomato male reproductive organs was conducted in our group. This investigation highlighted that many Hsfs are finely regulated in anther tissues at the pollen mother cell stage upon hs, and further supports the idea that Hps and Hsfs are important players in the hs response in pollen development. It should be noted that expression of Hsfs and Hsps occurs also in response to other stress factors and even in the absence of environmental stress stimuli, also during pollen formation [9,38,59,65,66,67,68,69,70]. It was suggested that during pollen development, Hsps function as molecular chaperones for the folding/refolding of proteins involved in meiosis and tetrad formation [60,63]. Dedicated mutants and transgenic plants will certainly help to unravel the functional significance of individual Hsfs and Hsps during anther and pollen development and in the protection of the male reproductive organs under hs. 

## 5. Proteomic Changes in Response to hs in Anther and Pollen Grains

Although proteomic technology in combination with genome sequence projects has remarkably increased our knowledge on plant proteomics [71,72], the information generated on developing pollen grains and anthers exposed to stress conditions is still scarce. Large-scale proteomic investigations have been performed at the stage of mature pollen grains [73,74]. In tomato, Sheoran *et al*. [67] and the following study of Lopez-Casado *et al*. [75] have confirmed that mature pollen grains store proteins for pollen germination and tube growth. At this stage, a considerable number of proteins is associated with energy conversion, intracellular trafficking, interactions with the maternal pistil tissues and defense mechanisms. On the contrary, few studies have analyzed the global changes of the proteome during microspore development [76,77]. In rice anthers at the microspore stage, the presence of several protein classes has been documented [77,78,79]. These proteins were classified as glycine rich proteins, hsps, proteosome-related proteins, post-translationally controlled proteins and chaperones. Changes in their profiles were reported in late developmental stages, with a considerable enrichment for proteins involved in pollen maturation and subsequent germination [76,77]. Useful information about protein changes associated with pollen fertility can also be retrieved from male sterility mutant analyses [80,81,82,83,84,85,86]. In Arabidopsis, failure of pollen development has been associated with the lack of adenine phosphoribosyltransferase (APRT) activity during microspore development [82]. In the male-sterile stamenless-2 mutant of tomato microspore degeneration was accompanied by the reduced activity of esterases and amylases [83,84]. The breakdown of pollen development in the environmentally induced male-sterile 7B-1 tomato mutant appeared also to correlate to certain proteins, particularly proteases and protease inhibitors, LRR-like proteins, stress related proteins and proteins with a role in cell division and lipid biosynthesis [85,86]. These proteins were supposed to function in the tapetum layer and pollen mother cells. Although it is widely reported that developing pollen grains and anthers are sensitive to environmental stress, few investigations have analyzed their global proteome changes under stress conditions [78,79,87]. In rice anthers, exposed to high temperatures at anthesis stage, it was observed that the hs protein profile is mainly characterized by the presence of protective proteins such as cold and heat shock proteins [87]. We have shown accumulation of HsfA2 and Hsp17-CII proteins in young anthers and mature microspores, and not in dry pollen upon hs [59]. Further large-scale investigations will certainly be useful to dissect hs response in developing pollen grains and anther tissues of tomato.

## 6. Metabolic Changes in Anthers and Pollen Grains upon hs

Within the anthers, the developing pollen grains are immersed in locular fluids containing nutrients such as sugars and lipids derived from the sporophytic tapetum tissue [17]. Early stages of pollen development are characterized by high metabolic changes and therefore, large amounts of sugars are mobilized from the source organs to the anther tissues [17,88,89]. At late stages, pollen maturation requires the accumulation of starch, which provides energy for subsequent germination and pollen tube formation [89]. Disturbances in sugar uploading and metabolism in the anthers can significantly impair pollen development and, thus, cause male sterility [88,90,91]. In tomato, changes in carbohydrate profiles were described under short and prolonged hs treatment of 32–36 °C, and were associated with the failure of tomato fruit set [92,93,94]. Major alterations in anthers were mainly observed for soluble sugars such as sucrose, fructose and glucose. Indeed, sucrose content increased in stressed anthers as compared to control tissues. Even though sugar metabolism is a complex pathway involving several routes, it was proposed that poor sucrose cleavage into soluble hexoses may be linked to the down-regulation of invertase genes occurring under hs [93]. In support of this, tomato RNAi-lines with silencing of the *Lin5* gene, encoding for an extracellular cell wall invertase, showed pollen malformation and abortion [95]. 

Massive accumulation of proline has also been reported in anthers and was associated with the protection of cellular structures, particularly during pollen dehydration [18,96,97,98]. In addition, proline abundance in pollen grains was thought to function as energy storage, for fueling the rapid demand of energy during pollen tube elongation [99]. Thus, pollen fertility can be markedly affected by proline changes in gametophytic and sporophytic tissues [100]. Interestingly, exposure to prolonged hs treatment of 32 °C/26 °C (day/night) disturbs proline uptake in tomato anthers at meiosis stage, probably due to the repression of specific transporters [18,93]. Whether this is related to hs-induced defects in early pollen development is still not known. 

Abortion of pollen under hs has also been associated with imbalances in polyamine content [101]. At 38 °C pollen germination and tube growth rates decrease considerably due to the reduction of spermidine and spermine contents [101]. Investigation of biosynthetic routes showed that the down-regulation of *S-adenosylmethionine decarboxylase* gene in the tapetum layer of tomato results in male sterility [102], suggesting that polyamines are also important metabolite involved in early developmental stages of pollen grains.

Together, these studies suggest that various metabolites are associated with tomato pollen performance under hs. However, knowledge in this area is very limited, suggesting that global metabolic analyses would markedly increase our understanding of the physiological response of pollen grains to hs. 

## 7. Genetic Variations in Pollen Thermotolerance

Species differ substantially in the tolerance of male fertility to high temperatures, and the same occurs for accessions within a species [103,104,105]. What can we learn from this natural variation? A number of studies has compared contrasting accessions and shown correlations of various characteristics (transcriptional, physiological, metabolic, developmental) with the heat tolerance trait. Bita *et al*. [39], for example, showed that a tolerant tomato line reacted less vigorously to hs than a sensitive one at the transcriptional level. Furthermore, Frank *et al*. [38] and Bita *et al*. [39] reported higher expression of Hsfs and Hsps in pollen and anthers, respectively, of a tolerant line compared to a sensitive line, already in the absence of hs, and four heat tolerant tomato lines were shown to maintain higher contents of starch and sucrose in the developing pollen grains than three heat sensitive lines [94]. Using a similar comparative approach, an increase of antioxidant enzyme capacity in flag leaf was observed in a heat tolerant rice variety (with better anther dehiscence upon heat), but not in a sensitive one [106] and in an analysis of several pairs of cowpea (*Vigna unguiculata*) contrasting in tolerance, heat tolerance at pod-set (as determined by pollen thermotolerance) was found to correlate with membrane thermostability [107]. However, all these experiments are based on a limited number of comparisons and, therefore, these studies cannot independently reveal the significance of identified correlations. To this end, experiments with direct manipulation of a characteristic in a single genetic background should be performed.

Accessions with contrasting levels of heat tolerance have also been used to determine the genetic basis of the trait using forward genetic approaches. Male fertility was seldom assessed directly in these studies, but because it has been shown to be the major determinant of fruit set success in many species, it is informative to look at this derived trait. A survey of studies in tomato, rice and cowpea, shows that in most cases the tolerance was based on multiple, small-to-medium effect loci, each explaining a few to maximum ~25% of the phenotype [108,109,110,111,112]. An exception to this may be a study in cowpea that identified a single, dominant heat tolerance gene [113]. Notably, reported QTL positions for the same species differ substantially between studies. This may largely be explained by the different germplasm that was used and indicate that the heat tolerance trait has many genetic determinants, which would agree with the finding that many different genetic manipulations can artificially increase heat tolerance [114]. However, QTL positions have even been found to vary between studies using the same tolerant parental accession [87,108], which points to a strong interaction with other genes in the background or to high sensitivity to the assay parameters. Regarding the first alternative, analyses by Opeña *et al*. [105] and Lin *et al*. [111] also suggested strong gene-interaction effects, as some hybrid offspring performed much better than either parent from a cross. Furthermore, some tolerance alleles have been reported to be derived from the sensitive parent of a cross [109,110]. Regarding the latter alternative, it is interesting to note that the tolerance traits of several heat tolerant green bean (*Phaseolus vulgaris*) varieties were highly specific to a particular temperature regime, *i.e.*, hot days and hot nights, versus hot days and cool nights [115].

Taken together, exploitation of natural variation has shed some light on general characteristics of heat tolerance traits and already led to breeding of a heat tolerant cowpea variety [116]. Unfortunately though, genes underlying the natural trait have not been identified as yet.

## 8. Conclusions and Future Perspectives

The development of functional pollen grains that are released at the appropriate stage to maximize the pollination process is a critical aspect of plant sexual reproduction. Omic-based profiling methods have considerably helped to understand the complexity of the regulatory signaling cascades involved in these processes and have led to the identification of many key genes. In view of the predicted changes in global temperatures and of the negative impact on sexual plant reproduction, it is urgent to develop appropriate strategies for improving crop productivity under environmental constraints. As tomato is an important crop often exposed to temperature fluctuations during reproduction, the understanding of molecular mechanisms involved in hs response is a hot-topic. Genome-wide analyses have started to provide some candidate genes involved in thermotolerance, which can now be characterized in more detail. Future efforts should also be devoted to generate and integrate-omic data from metabolic and proteomic analyses and to combine them with results retrieved from genetic studies. Such knowledge could become important for the identification, selection and transfer of superior alleles and for generation of thermotolerant lines via conventional breeding or using biotechnology strategies.
